# Disadvantaged Patients, Health Care Providers, and Natural Disaster Preventive Planning

**DOI:** 10.1055/s-0043-1778069

**Published:** 2024-01-31

**Authors:** Ahmed Alnajar

**Affiliations:** 1 Division of Cardiothoracic Surgery, DeWitt Daughtry Department of Surgery, University of Miami Miller School of Medicine, Miami, Florida, United States; 2 Division of Biostatistics, Department of Public Health Sciences, University of Miami Miller School of Medicine, Miami, Florida, United States

The region including Türkiye and Syria was left devastated after multiple earthquakes in February 2023 that struck Türkiye's southern province of Hatay and Northwest Syria (up to 7.8 Richter), which left 40,000 people dead, many more injured, and at least 1.5 million homeless in Türkiye. 1 Some authors attempted to address the effects of the disaster, 2 which included the revelation that “the earthquakes have also put a huge strain on the medical workforce, as doctors and nurses have had to work long hours to treat injured people and manage the crisis. This strain has further exacerbated the preexisting workforce shortages and burnout of Türkiye's health care system.” In our experience dealing with natural disasters, we have implemented a strategic response to hurricanes that involve staff and patients and preventive plans. 3

Natural disasters, such as earthquakes and their following aftershocks, can affect vulnerable people the most. Türkiye is especially vulnerable, as it hosts the largest number of refugees displaced from Syria. Thus, it becomes crucial to assess the differential impact of the earthquakes on vulnerable populations, particularly those with limited access to health care resources. Furthermore, a comprehensive evaluation of the preparedness and response mechanisms within the health care system is essential to understand potential shortcomings and areas for improvement in addressing the needs of disadvantaged patients during such catastrophic events. Collaborative efforts between health care authorities, policymakers, and disaster management agencies are imperative to ensure that future disaster preventive planning incorporates equitable access to health care services and prioritizes the most vulnerable segments of society. Furthermore, both immediate response and preventive plans should have special considerations for refugees, who may be unable to evacuate. These plans can help populations avoid traumatizing exposure and access specialized centers that may likely decrease the health care burden, improve disaster-mitigating efforts, and yield better long-term outcomes, as seen in patients with preexisting diseases. 4 5 6

We agree that comprehensive and well-coordinated plans are necessary for the management of the crisis. However, preventive action is also necessary. As in the prehurricane plan, 3 earthquake planning should be set as a regular preventive action. There should be a training session for supporting the health care team and patient care provider in emergency management of the situation—not only in the affected areas, but also in the traveling emergency response groups—focused on vulnerable people. There were numerous instances of insufficient care and services for vulnerable populations that underscore the immediate necessity for focused interventions and enhanced disaster preparedness planning.

1. **Access to medical care** : Disadvantaged communities, often residing in remote or underserved areas, faced significant challenges in accessing medical care due to disrupted transportation routes, damaged health care facilities, and overwhelmed health care personnel. 1 2 7 This resulted in delayed or insufficient medical attention for cancer patients, and those with injuries and infections. 7
2. **Shelter and sanitation** : Vulnerable populations, including the elderly, children, and disabled individuals, encountered difficulties in finding suitable temporary shelters that catered to their unique needs. 1 Lack of accessible sanitation facilities in these shelters further exacerbated health risks, potentially leading to the spread of infectious diseases.
3. **Medical supplies and medications** : Shortages of essential medical supplies, medications, and equipment hindered the ability to provide adequate treatment to vulnerable patients. Those relying on continuous medical interventions, such as chronic diseases (e.g., diabetes, heart failure, cancer), 3 5 are prone to disruptions that jeopardize their well-being.
4. **Communication barriers** : Refugees, in particular, often experience language barriers or limited access to information about available health care services, emergency contacts, and relief efforts. This hinders patients' ability to seek help promptly and make informed decisions during crises.
5. **Specialized care** : Vulnerable populations requiring specialized medical care, such as individuals with disabilities or mental health conditions, 8 9 encountered difficulties in accessing appropriate services tailored to their specific needs. The lack of trained personnel and appropriate facilities further compromised their care.
6. **Psychosocial support** : Earthquake can take a toll on the mental well-being of many vulnerable individuals, with limited access to psychosocial support services. 9 This include counseling, therapy, and emotional assistance for those grappling with trauma, grief, and anxiety.
7. **Equitable resource allocation** : The distribution of resources and aid may not have been equitable, potentially leaving disadvantaged populations underserved in terms of food, water, health care, and other essential supplies, which could leave patients in extremely cold temperatures without drinking water, or electricity. 1

Since monitoring of competency for response to disasters is necessary, thus, it is critical for governments, as well as medical and disaster-relief societies, to apply integrated risk reduction and management and disaster preparedness measures for both physical and mental issues if the region is to survive the many disasters that are sure to come.

In conclusion, collaborative efforts should help mitigate further catastrophes through preventive earthquake-response planning. Providing adequate training for physical injuries and psychological support is of paramount importance, especially for refugees.
